# RADx-UP Testing Core: Access to COVID-19 Diagnostics in Community-Engaged Research with Underserved Populations

**DOI:** 10.1128/jcm.00367-23

**Published:** 2023-07-03

**Authors:** Shanti Narayanasamy, Timothy H. Veldman, Mark J. Lee, William A. Glover, L. Gayani Tillekeratne, Coralei E. Neighbors, Barrie Harper, Vidya Raghavan, Scott W. Kennedy, Miranda Carper, Thomas Denny, Ephraim L. Tsalik, Megan E. Reller, Warren A. Kibbe, Giselle Corbie, Michael Cohen-Wolkowiez, Christopher W. Woods, Cathy A. Petti

**Affiliations:** a Division of Infectious Diseases, Department of Medicine, Duke University, Durham, North Carolina, USA; b Hubert-Yeargan Center for Global Health, Duke University, Durham, North Carolina, USA; c Duke Global Health Institute, Durham, North Carolina, USA; d Department of Pathology, School of Medicine, Duke University School of Medicine, Durham, North Carolina, USA; e North Carolina State Laboratory of Public Health, North Carolina Department of Health and Human Services, Raleigh, North Carolina, USA; f Duke Clinical Research Institute, Duke University, Durham, North Carolina, USA; g Duke Human Vaccine Institute, Duke University, Durham, North Carolina, USA; h Department of Biostatistics and Bioinformatics, Duke University School of Medicine, Durham, North Carolina, USA; i Duke Cancer Institute, Duke University School of Medicine, Durham, North Carolina, USA; j Center for Health Equity Research, University of North Carolina, Chapel Hill, North Carolina, USA; k Department of Social Medicine and Department of Medicine, University of North Carolina, Chapel Hill, North Carolina, USA; l Department of Internal Medicine, University of North Carolina, Chapel Hill, North Carolina, USA; m Department of Pediatrics, Duke University School of Medicine, Durham, North Carolina, USA; n Healthspring Global Inc., Bradenton, Florida, USA; Vanderbilt University Medical Center

**Keywords:** RADx-UP, COVID-19, diagnostics, equity, underserved communities

## Abstract

Research on the COVID-19 pandemic revealed a disproportionate burden of COVID-19 infection and death among underserved populations and exposed low rates of SARS-CoV-2 testing in these communities. A landmark National Institutes of Health (NIH) funding initiative, the Rapid Acceleration of Diagnostics-Underserved Populations (RADx-UP) program, was developed to address the research gap in understanding the adoption of COVID-19 testing in underserved populations. This program is the single largest investment in health disparities and community-engaged research in the history of the NIH. The RADx-UP Testing Core (TC) provides community-based investigators with essential scientific expertise and guidance on COVID-19 diagnostics. This commentary describes the first 2 years of the TC’s experience, highlighting the challenges faced and insights gained to safely and effectively deploy large-scale diagnostics for community-initiated research in underserved populations during a pandemic. The success of RADx-UP shows that community-based research to increase access and uptake of testing among underserved populations can be accomplished during a pandemic with tools, resources, and multidisciplinary expertise provided by a centralized testing-specific coordinating center. We developed adaptive tools to support individual testing strategies and frameworks for these diverse studies and ensured continuous monitoring of testing strategies and use of study data. In a rapidly evolving setting of tremendous uncertainty, the TC provided essential and real-time technical expertise to support safe, effective, and adaptive testing. The lessons learned go beyond this pandemic and can serve as a framework for rapid deployment of testing in response to future crises, especially when populations are affected inequitably.

## TEXT

The COVID-19 pandemic highlighted longstanding structural inequities in society. Barriers to health care access and resources, structural racism, and historical discrimination have resulted in a disproportionate rate of illness and death among minority and underserved communities (1–3). However, these issues are not new. Historically marginalized communities in the United States (e.g., communities of color, migrant communities, incarcerated individuals, and those living in poverty) have been underrepresented in clinical trial recruitment (4), health technology innovation (5), and testing access (6–8). Despite an increased risk of infection and death from COVID-19, high-risk communities are less likely to be tested for infection (9, 10). Current approaches to testing have been developed and implemented primarily in well-resourced populations, but low testing rates in some populations are often community specific and require a tailored approach (11, 12). Even when barriers to testing are well elucidated, translating that knowledge to action is critical to breaking down those barriers. Novel, community-engaged approaches are key to addressing COVID-19 disparities among these different populations (13).

In the manuscript, we first describe the Rapid Acceleration of Diagnostics-Underserved Populations (RADx-UP) program and the RADx-UP Testing Core (TC). Second, we discuss the evolution of the pandemic and how this impacted diagnostic testing imperatives. Finally, we elaborate on the shifting landscape of the pandemic that changed the TC’s focus and the subsequent establishment of four strategies (Table 1) that enabled the TC to support the deployment of large-scale testing for community-based research in underserved populations.

**TABLE 1:** Strategies employed by the RADx-UP Testing Core to support large-scale testing for community-based research with underserved populations during the COVID-19 pandemic

No.	Strategy	Description
1	Individualize test strategies for each unique study setting and population	Adaptive and agile standardized assessment tools are needed to support the individualized testing goals of studies. Monitoring frameworks should be put in place to continuously monitor test performance and use, and testing strategies should be responsive to community feedback.
2	Make knowledge accessible and digestible	Testing and regulatory expertise needs to be disseminated to investigators in real time and through accessible methods to support study needs.
3	Build a resilient and adaptable research culture for unexpected events	Build a research culture that is flexible, resilient, and able to pivot to respond to changes in a pathogen’s epidemiology, participant recruitment, and test kit manufacturing supply.
4	Invest in capacity building to create a runway for the future	Invest in capacity building for enabling underserved populations to address future public health challenges through access to health care advancements and technologies that are community driven.

## THE RADX-UP PROGRAM

In September 2020, the National Institutes of Health (NIH) committed $1.4 billion to accelerate the innovative development and implementation of COVID-19 testing (14). RADx-UP (15), part of this initiative, represents the single largest investment in health disparities and community-engaged research in the history of the NIH. NIH established RADx-UP to reduce disparities in COVID-19 testing by funding community-based research studies in the United States. As of 1 August 2022, RADx-UP had funded 127 projects across the United States, including 75 studies that provide SARS-CoV-2 testing directly to participants (see Fig. S1 in the supplemental material), with a total expected enrollment of >1.5 million participants.

## THE RADX-UP TESTING CORE

The RADx-UP TC is one of three RADx-UP Coordination and Data Collection Center (CDCC) pillars, alongside the Community Engagement and Data Science & Biostatistics cores. Each pillar provides its relative subject matter expertise within RADx-UP CDCC, and as such, the TC provides essential technical expertise and scientific guidance on COVID-19 diagnostics to the NIH, the CDCC, and community-based investigators and their community partners to solve testing challenges (16). The magnitude and diversity of expertise of the TC are substantial. The knowledge domains and experience of TC members span clinical and public health microbiologists, infectious disease physicians, research scientists, regulatory experts, and leaders in the diagnostic industry. This assembly of experts enables the TC to provide a deep understanding of the diagnostic, laboratory, and regulatory landscape to implement guidance at record speed during a pandemic. The central role of the TC is to support and tailor each study’s SARS-CoV-2 testing goals for their unique populations, test settings, and needs. This support includes (i) a critical review of protocols to advise on optimal testing strategies that meet project goals, (ii) knowledge distribution about different COVID-19 test targets (viral antigen, viral nucleic acid, immune response) and compliance requirements, and (iii) opening channels for procurement of test kits, sample collection devices, reagents, and medical equipment for testing.

The TC held its first meeting in October 2020, at which time 38 projects had been awarded. The TC recognized the need to balance process-driven policies and procedures for testing guidance while maintaining an agile response to changes in market dynamics, advances in testing technologies, regulation and compliance requirements, emergence of viral variants, and the development of vaccines. Early in the pandemic, the TC realized that scientific guidance in testing could not be a static process, and research support required nonlinear analyses of the testing situation for each project. As such, adaptation and iteration underpinned key behaviors of the TC.

## EVOLUTION OF A PANDEMIC: TESTING AND VACCINE WAVES ARE AS IMPORTANT AS VIRAL WAVES

### Period 1: early testing (March 2020 to January 2021).

**FIG 1:**
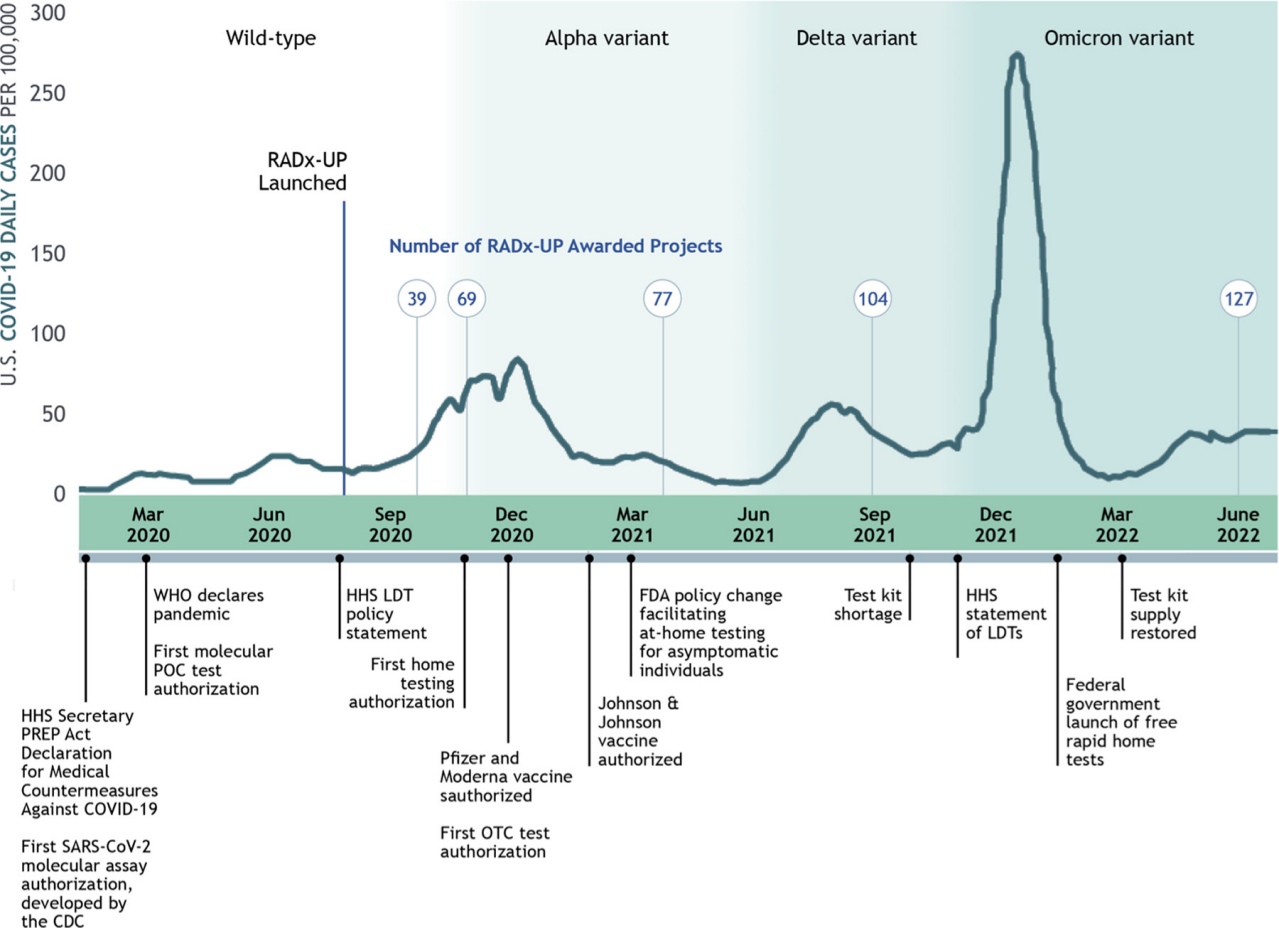
Timeline of RADx-UP Testing Core activities during the COVID-19 pandemic. CDC, Centers for Disease Control and Prevention; FDA, U.S. Food and Drug Administration; HHS, U.S. Department of Health and Human Services; LDT, laboratory-developed tests; OTC, over the counter; POC, point-of-care; PREP, public readiness and emergency preparedness; WHO, World Health Organization.

Early in the pandemic, testing was implemented through two processes, the U.S. Food and Drug Administration’s (FDA’s) emergency use authorization (EUA) and the FDA’s enforcement discretion of laboratory-developed tests (LDTs) (Fig. 1). In 2004, legislation was enacted to establish the EUA in response to threats of bioterrorism and naturally occurring emerging infections (17). Since then, EUAs have been issued for influenza, Middle East respiratory syndrome coronavirus, Ebola virus, and Zika virus (18). The COVID-19 pandemic sparked an unprecedented expansion of tests authorized under EUA for *in vitro* diagnostic products (19). LDTs, in existence since 1976 and administered by the Centers for Medicare and Medicaid Services under the Clinical Laboratory Improvement Amendments (CLIA) (20), were also developed rapidly. During the early testing period of the pandemic, we supported study investigators in understanding and balancing policies for EUA testing with those for LDTs, particularly regarding the regulatory aspects of allowable specimen collection and testing locations.

### Period 2: viral variants, vaccination, and serological testing (February 2021 to September 2021).

Widespread and broadly available vaccination from January 2021 changed the landscape for SARS-CoV-2 testing. As vaccination increased, the incidence of COVID-19 infection decreased, as did the rate of testing. Studies that were enrolling participants experienced a decline in recruitment, and the rationale for testing required greater emphasis in the setting of increasing vaccine-related immunity. Many studies capitalized on existing testing infrastructure to bring vaccines to underserved populations. This period was also marked by an increased interest in testing for immune response to SARS-CoV-2 to assess the seroprevalence of individuals with prior infection and prior vaccination. Community investigators questioned test performance with respect to viral variants. Finally, rapid swings in the demand for testing influenced by vaccination and viral variants influenced the ability of test manufacturers to forecast testing supplies, leading to episodic testing shortages. The dynamism between virus and vaccination led to the programs developed under strategies 1 and 2, described below (Table 1).

### Period 3: home-based testing (October 2021 to March 2022).

The demand for home-based sample collection and rapid testing increased with the arrival of the Delta and Omicron SARS-CoV-2 variants. Home testing was viewed as a way to enable safer family gatherings, remain in school, return to the office, and allow for large public gatherings. However, as the incidence of COVID-19 infection increased, demand for home-based testing rapidly outstripped test supply. These unexpected changes coincided with the relaxation of stringent public health measures as the primary tools to control transmission. This period illustrated the importance of resilience in community-based research (strategy 3) and the importance of building innovative laboratory capacity for future community-driven research (strategy 4) (Table 1).

## RADX-UP TESTING CORE GUIDING STRATEGIES

### Strategy 1: individualize test strategies for each unique study setting and population.

**FIG 2:**
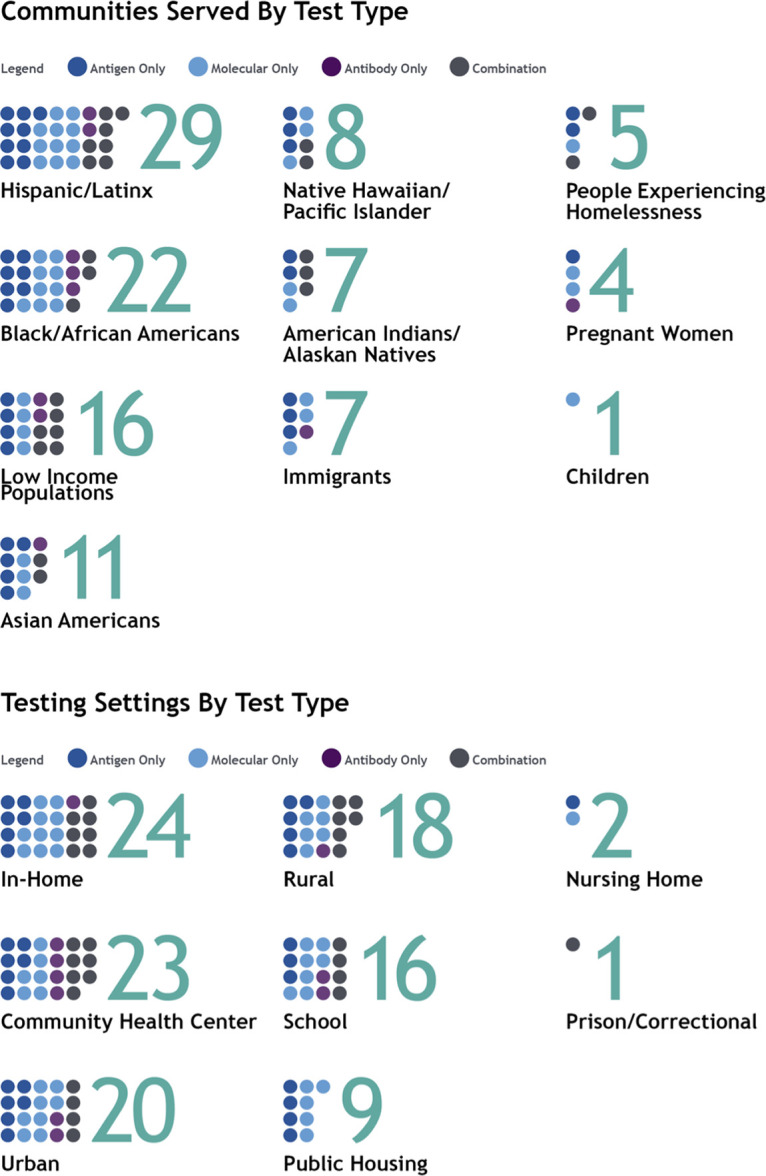
RADx-UP communities and settings by test type (projected estimates for studies testing participants directly).

RADx-UP aims to conduct research on how best to ensure equity in access to community-centered SARS-CoV-2 testing among underserved communities. RADx-UP investigators are engaging populations that include racial and ethnically diverse communities, older adults, children, sexual and gender minorities, incarcerated populations, immigrants, people who use drugs, people with disabilities, people experiencing housing insecurity, people living in rural or geographically isolated regions, and pregnant women. The study settings vary and include rural, urban, tribal lands, schools, long-term care facilities, public housing, community health centers, in-home, and prisons/correctional facilities. As of August 2022, 75 studies have used NIH funds to provide SARS-CoV-2 testing directly to participants (see Fig. S1 in the supplemental material). Based on the projected enrollment of these studies, the following populations were represented: 39% (29/75) Hispanic/LatinX, 29% (22/75) Black, 15% (11/75) Asian, 9% (7/75) Alaskan native/Tribal Nations, 11% (8/75) Hawaiian/Pacific Islander, and 21% (16/75) low income (Fig. 2). The following test settings were projected by studies: 31% (23/75) community health centers, 24% (18/75) rural communities, 21% (16/75) schools, 32% (24/75) in-home, and 12% (9/75) public housing (Fig. 2). No significant trend was observed for type of test (e.g., molecular, antigen), test population, or setting. We developed adaptive tools to support studies to ensure the safe and effective rollout of COVID-19 testing across these different study populations and test settings.

**(i) Implement adaptive, agile, standardized assessment tools.** The TC developed two standardized assessment tools to support testing by RADx-UP studies. The Testing Assessment Quality Management Tool (assessment tool) (Fig. S2) ensured that the research teams’ testing strategies met study goals for their target populations. The tool was based on Good Clinical Practice and Good Laboratory Practice Guidelines for Nonclinical Laboratory Studies (21, 22). For each research grant, the study protocol, RADx-UP intake survey, and institutional review board (IRB) documents were carefully reviewed to extract essential elements. We identified information about the study setting, target population, specimen collection plan, intended use of testing (diagnosis, screening, contact tracing, or surveillance), and testing location to identify potential operational and regulatory challenges. The TC used the assessment tool to create brief reports (Fig. S3) that provided specific guidance to each study for testing deployment. The brief reports were updated and amended over time to respond to the dynamic regulatory and policy environment and the changing testing strategies of studies.

Two projects in the early part of the COVID-19 pandemic demonstrate the value of standardized assessment tools and brief reports in supporting research teams. In 2020, community-based researchers developed a project to understand the utility of point-of-care (POC) testing for workplace surveillance in a group of rural coal miners (23). The assessment tool found that the team planned to use lateral flow antigen testing under health care supervision in symptomatic and asymptomatic miners before their work shifts. When the study was developed, federal and state guidance around antigen testing for COVID-19 surveillance and testing without health care supervision was not established. The ability to rapidly obtain a CLIA waiver was also not available. In partnership with the study team, the TC facilitated the process to obtain a CLIA waiver for POC testing—an unfamiliar process for the investigative team. To assist deployment of POC antigen testing, the TC furnished a recently released, and not yet widely adopted, CDC guidance document to ensure safe deployment of antigen testing for asymptomatic individuals through serial antigen testing and, when applicable, confirmatory testing with molecular methods (24). The CDC guidance document became a core tool until FDA EUA antigen tests for asymptomatic individuals were available.

A second project commencing early in the pandemic served as another exemplar of the importance of agile, adaptive assessment tools. Study investigators were studying the incidence of COVID-19 among incarcerated persons and correctional staff within prisons and jails across four states. The setting presented unique logistical and operational challenges, particularly around optimal specimen type (e.g., nasal or saliva) and collection by a health care worker. In collaboration with the TC, the research team identified a POC antigen test for nasal specimens and a PCR saliva-based collection, both of which were authorized for self-collection with or without health care supervision. The TC also supported POC testing under a CLIA waiver by providing the study team with resources for training test operators to consistently and reliably perform POC tests (as specified in CLIA regulations) that could be adopted in a resource-constrained setting.

**(ii) Develop frameworks to continuously monitor test performance and use.** The TC stayed up to date with changes in test performance and use through the emerging literature (preprint and published), FDA press releases and warning letters on test performance, and abstracts presented at conference proceedings. This information was rapidly synthesized and disseminated to study investigators to support their testing choices. In many urban areas, access to testing was not uniform across the city, with large “testing deserts,” defined as an area that is at least 10 miles from a testing center, in lower socioeconomic and racially and ethnically segregated neighborhoods (25). One RADx-UP study sought to increase COVID-19 testing in public housing buildings, an area with high COVID-19-related mortality but low testing uptake. The target populations were largely LatinX and Black, had limited English proficiency, and commonly included intergenerational, high-density households. Household transmission was a key driver of COVID-19 cases in their locale, so a low-cost, sustainable solution to provide the greatest benefit to the public housing community was imperative (26). While working with this study team, we identified several other research proposals that faced similar challenges. Home-based antigen testing was limited to telehealth during this time period, and several investigators were concerned about limited internet access and the accuracy of antigen tests. Molecular-based tests for home use during this early phase of the pandemic were restricted to home specimen collection shipped to a centralized laboratory for molecular testing. With this approach, some researchers felt that the benefits of greater accuracy using PCR tests were eclipsed by participants’ limited access to mailboxes, high costs of testing, and slow time to results. To reduce cost and ensure rapid results, a few investigators proposed home-based specimen collection with subsequent testing using an LDT PCR test at their laboratories. However, this approach was not permitted by the FDA’s LDT policy for SARS-CoV-2 (27).

The NIH policy requiring FDA emergency use authorizations for tests presented an additional challenge. While this policy was an important stipulation to ensure the quality and accuracy of tests as well as to protect underserved communities with a history of research exploitation, it limited testing options for community-based investigators who often had ready access to LDTs but had challenges in procuring FDA EUA tests during periods of high demand. The TC helped study teams surmount these challenges by facilitating a pivot to other testing strategies.

**(iii) Testing strategies responsive to community feedback.** RADx-UP research teams sought sustainable, low-cost, and accessible testing solutions for their communities. For some studies, participants had expressed reluctance in using uncomfortable or onerous testing methods, such as nasopharyngeal and nasal swabs. Saliva testing was a way to surmount this barrier. One study investigated saliva testing in the school setting for students with intellectual and developmental disabilities, as nasal testing was not feasible in their participant population (28). The TC supported the study’s decision to use FDA EUA saliva testing through participant self-collection at home and under supervision at school. This testing approach provided a safe and acceptable collection method in children and engaged a population that is otherwise underrepresented in clinical research.

The TC also received feedback about the urgent need for testing instructions in Spanish. In response, our procurement team worked with test manufacturers to identify those with FDA EUA labeling in languages other than English. Also, some study teams sought tests that did not require the internet, were suitable for participants with low literacy and numeracy skills, and did not require the participant to read their results. Following this feedback, we developed a repository of test options that could be queried and filtered by key performance features, such as test target (important for serology testing), internet requirements for testing, available language translations, and health care provider-supported test result interpretation.

### Strategy 2: make knowledge accessible and digestible.

The TC observed that digestible knowledge about *in vitro* testing must be communicated to research teams. The TC embraced current concepts in adult learning to provide information across multiple modalities, such as written reports, visual charts, oral online presentations, emails, flyers, and one-to-one video meetings. We prioritized meeting with individual study teams, often monthly, to address the testing complexities unique to their target population and study settings. Through these face-to-face meetings, we identified common themes across multiple studies. In response, the TC developed quick reference guides (QRGs) on POC testing, home testing, saliva testing, and antibody testing (29). The purpose of the QRGs was to provide investigators with highly digestible, concise summaries of test characteristics for FDA EUA testing kits. This approach was data driven, and QRGs were updated in real time and made available on the RADx-UP website. These invaluable guides also provided information on recalls, EUA revocations, and, at the height of testing shortages, the availability of testing kits. We also used email informationals to update study teams on important emerging topics. This enabled us to inform teams rapidly and simultaneously about supply chain delays, challenges with test kit performance (false positives and negatives), and regulatory changes.

Another forum for collaboration and learning was among RADx-UP studies themselves. Through monthly, program-wide meetings, the TC spotlighted current issues or topics of particular interest in testing to a large investigator audience. We encouraged and facilitated peer learning by connecting studies experiencing similar testing challenges. These cross-study engagements were opportunities to share best practices in result reporting, use of centralized laboratory testing to identify viral variants, and use of sequencing-based technologies to study the evolution of viral variants.

Finally, the TC supported investigators who sought to understand the evolving landscape for non-EUA novel diagnostic technologies. We leveraged existing partnerships with Arizona State University to provide a web repository of current and emerging technologies in COVID-19 testing (30). The website supports users in matching test kits with their required regulatory status, diagnostic targets, collection methods, and test processing locations.

### Strategy 3: build a resilient and adaptable research culture for unexpected events.

Clinical research supervised through an IRB has historically followed a linear process. The study design is clearly defined and approved by the IRB, and any changes to the study require formal amendments and IRB approval. During the pandemic, evolving testing tools, changing viral dynamics, test kit shortages, and fluctuating patterns in participant engagement challenged this linear process.

During the pandemic, the TC supported study teams in negotiating many unexpected changes, underscoring the importance of specialized and diagnostic expertise during this transition period. SARS-CoV-2 incidence fluctuated throughout the United States across population groups, test settings, and geographic areas. In 2020, many RADx-UP research teams were preparing their studies, obtaining IRB approvals, procuring tests, and organizing other logistics while COVID-19 case incidence was high. As these studies were launching in 2021, investigators began to face recruitment challenges. COVID-19 infections plummeted with increased immunity and vaccinations, and community interest in testing rapidly declined. Many investigators pivoted to vaccine engagement and refined their testing strategies to study seroprevalence. In response, the TC employed microlearning techniques, including targeted content through blogs and short reports, to support investigators’ knowledge acquisition and to guide researchers through testing protocol changes and IRB amendments.

### (i) Procurement support is essential to navigating shifting supply and demand.

The RADx-UP test procurement team was pivotal in forging communication channels with testing manufacturers and suppliers. As the EUA approval process became more streamlined, the team established a curated vendor list of >80 suppliers and served as a liaison between suppliers and studies. The importance of these relationships was underscored during periods of testing kit supply shortages. When the Delta and Omicron variants became the dominant U.S. SARS-CoV-2 variants in 2021 and 2022, shortages in the supply chain for FDA EUA at-home tests posed a challenge for many investigators. The TC was able to capitalize on preexisting relationships with manufacturers and other governmental bodies to emphasize the scientific mission of RADx-UP and encourage suppliers to meet the research needs for testing in underserved communities. One major test kit manufacturer provided home antigen test kits for free to seven RADx-UP projects. The TC also approached the Department of Defense, which released and donated over 90,000 test kits to several projects.

### Strategy 4: invest in capacity building to create a runway for the future.

Access to health care advancements and technologies is challenging for underserved communities during national emergencies. The RADx-UP Rapid Research Pilot Program (RP2) was established to help address this disparity. Pilot projects were awarded through the TC to provide an opportunity to study novel EUA-authorized POC methods, home collection devices, and at-home testing. RP2 investigators work in underserved communities and collaborate with technology innovators to improve access and uptake of innovative technologies. Sixteen RP2 projects have been approved through August 2022, with plans to fund nine more. One RP2 project leverages crowdsourcing of innovative technologies that will help encourage community engagement and amplify the project’s ability to identify novel test tools. Another project uses crowdsourcing and “designathons” to engage youth and teen populations, often overlooked or considered superspreaders, to create innovative strategies for participatory self-testing. A third awardee is studying the use of a mobile laboratory to understand the feasibility of providing convenient, accessible testing to local homeless and transitional populations. These innovative methods show great promise and could be scaled up to provide sustainable, widely accessible testing and essential tools for managing future pandemics in underserved communities.

## CONCLUSION

RADx-UP has shown that increased access and uptake of testing among underserved populations can be successfully accomplished during a pandemic when community-based research is supported with tools, resources, and the wide expertise of a centralized, testing-specific coordinating center. In the setting of tremendous uncertainty and a rapidly evolving public health crisis, the TC provided essential and real-time technical expertise to support safe, effective, and adaptive testing. The lessons learned go beyond this pandemic and can serve as a framework for rapid deployment of testing both on a large scale and at the community level, specifically for future health crises that invariably affect populations inequitably. The COVID-19 pandemic acutely highlights the importance of diagnostic testing expertise and dissemination of knowledge. Testing knowledge that is readily accessible, digestible, continuous, and responsive supports investigators in navigating the shifting policy, regulatory, and clinical landscapes. Innovation and rapid adaptation will remain the cornerstone of progress as the TC now navigates solutions to link test results to care interventions, such as oral antivirals and other therapeutics. Community-based research is critical to elucidating the unique barriers and challenges faced by specific populations, and results from RADx-UP studies will better inform local, state, and federal public health policies to optimize testing strategies and access.
