# Supplementary material for: RADx-UP Testing Core: Access to COVID-19 Diagnostics in Community-Engaged Research with Underserved Populations
Source: J Clin Microbiol. 2023 Jul 3;61(8):e00367-23. doi: 10.1128/jcm.00367-23 (PMC10446854; doi:10.1128/jcm.00367-23)
Supplement: Supplemental file 1 — Supplemental material. Download jcm.00367-23-s0001.docx, DOCX file, 0.8 MB [file jcm.00367-23-s0001.docx]

**Supplementary appendix**

**Fig S1** RADx-UP map of studies providing SARS-CoV-2 testing directly to participants, August 2022 ………………….……………… 2

**Fig S2** RADx-UP testing assessment tool …………………………………………………… 3

**Fig S3** RADx-UP brief report template ……………………………………………….…...… 4

Fig S4. Roles of Testing Core Members and Activities of the Testing Core…………………. 5

**Supplemental FIG S1** RADx-UP map of studies providing SARS-CoV-2 testing directly to participants, August 2022**
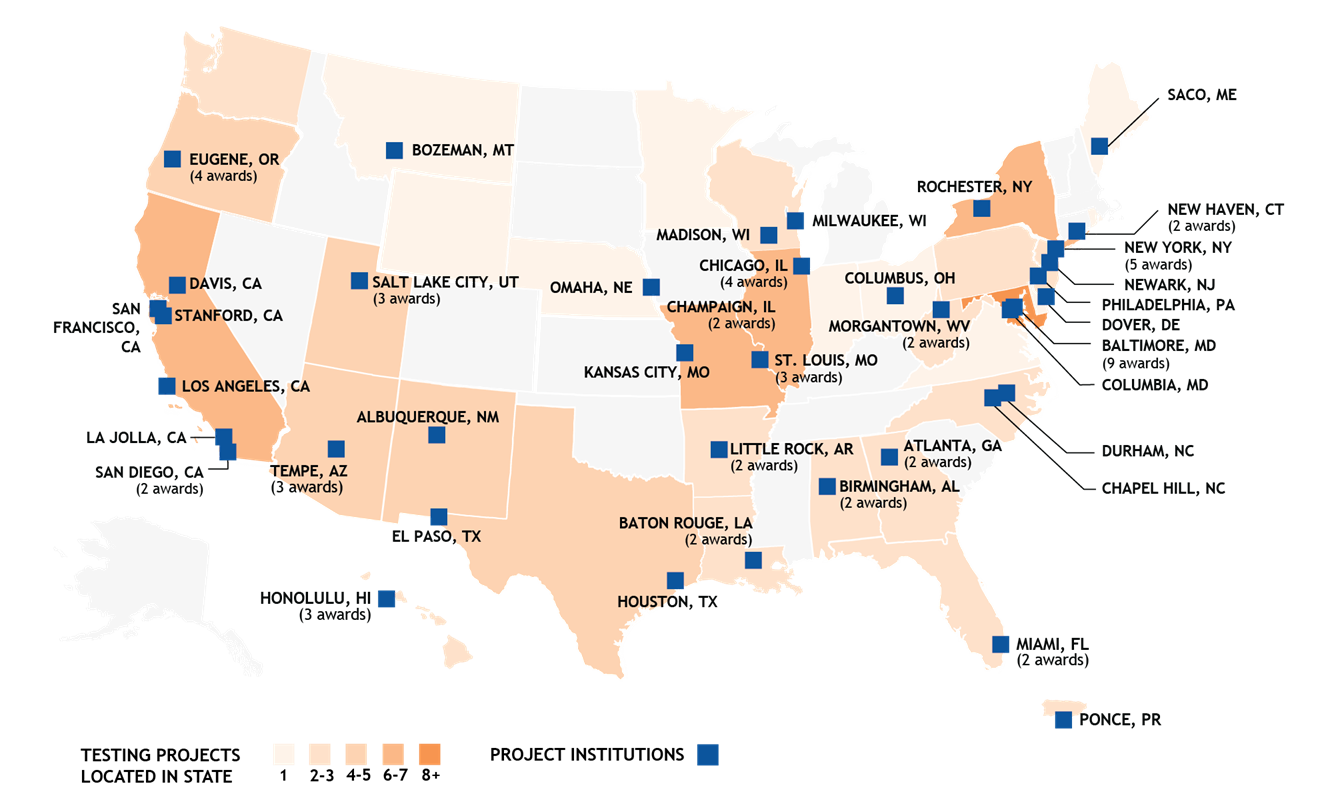
**


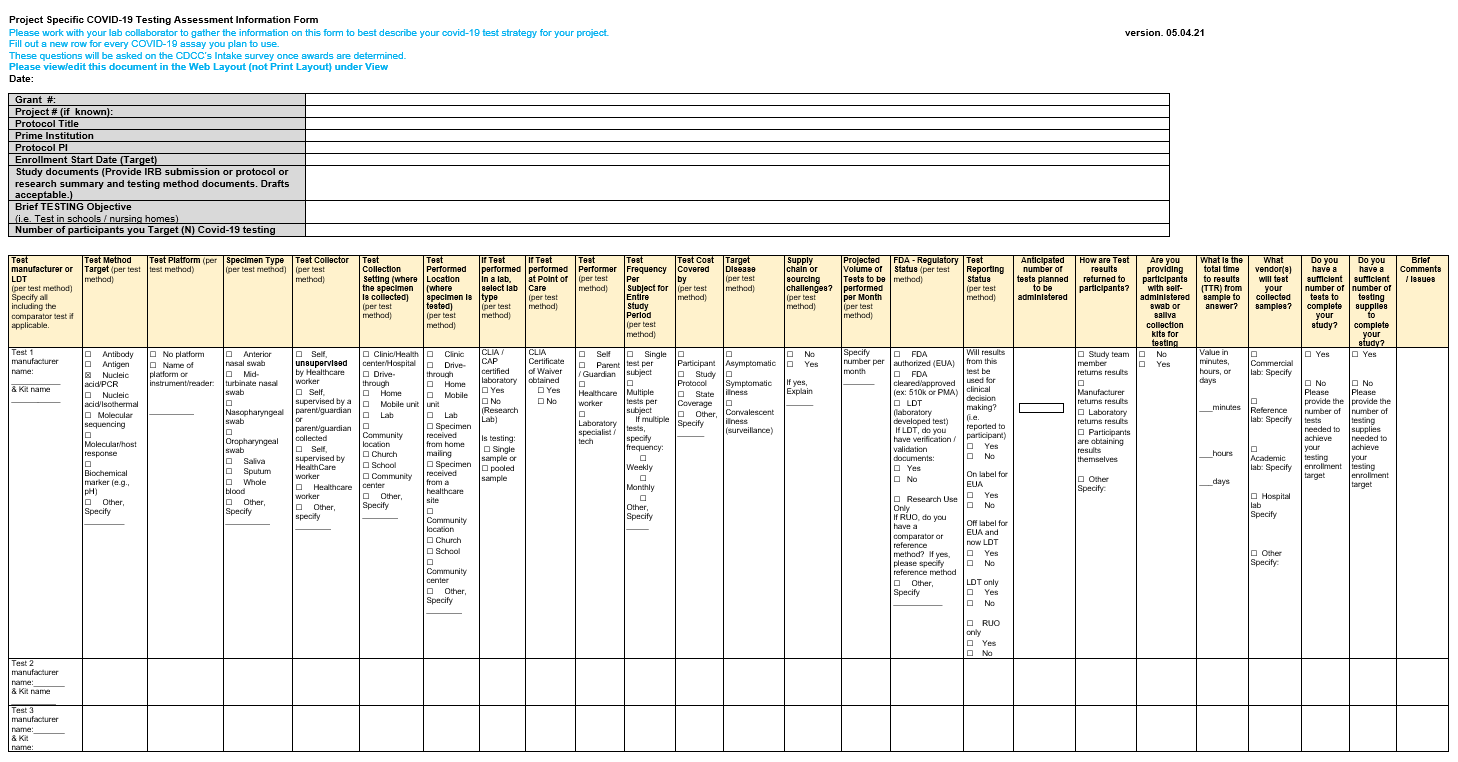
**Supplemental FIG S2** RADx-UP testing assessment tool

**Supplemental FIG S3** RADx-UP brief report template


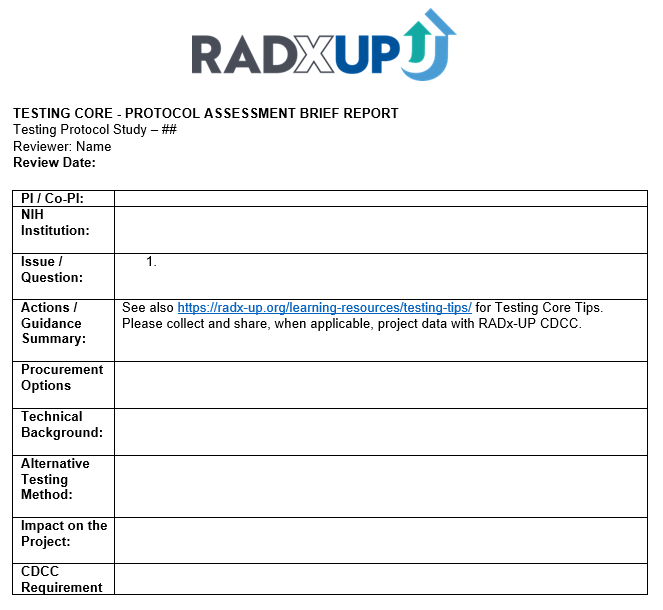


**Supplemental FIG S4** Roles of the Testing Core Members and Activities of the Testing Core

| **Roles of the Testing Core Members** |
| --- |
| COVID-19 test design and research and development expertise |
| Expertise in testing validation and verification |
| CLIA and FDA regulatory expertise |
| Expertise in public health, high throughput, and CLIA laboratory testing |
| **Activities of the Testing Core** |
| Provide technical assistance to study investigators, CDCC and NIH regarding COVID-19 test selection and implementation |
| Develop, curate, and maintain a repository of emerging technologies in COVID-19 related diagnostics and disseminating diagnostic technology assessments |
| Develop, manage, and evaluate the Rapid Pilot Studies in close collaboration with NIH |
| Provide vendor and supply chain management and advice for study investigators and the CDCC |
| Periodic assessments of project progress and advise on testing-related factors to support studies |

**Abbreviations**: CLIA *Clinical Laboratory Improvement Amendments*; FDA *U.S Food and Drug Administration*; CDCC *RADx-UP Coordination and Data Collection Center*; NIH *National Institutes of Health*.
